# Sex-Based Differences in Gut Microbiota Composition in Response to Tuna Oil and Algae Oil Supplementation in a D-galactose-Induced Aging Mouse Model

**DOI:** 10.3389/fnagi.2018.00187

**Published:** 2018-06-26

**Authors:** Hongyan Zhang, Zhaoyang Wang, Yanyan Li, Jiaojiao Han, Chenxi Cui, Chenyang Lu, Jun Zhou, Lingzhi Cheong, Ye Li, Tingting Sun, Dijun Zhang, Xiurong Su

**Affiliations:** ^1^School of Marine Science, Ningbo University, Ningbo, China; ^2^College of Agriculture and Life Sciences, Cornell University, Ithaca, NY, United States

**Keywords:** tuna oil, algae oil, aging, gut microbiota, sex-based differences

## Abstract

Our previous work indicated that a mixture of tuna oil and algae oil treatment in male mice effectively relieved D-galactose (D-gal)-induced aging and resulted in gut microbiota alterations, and that the best anti-aging effects were observed for a tuna oil to algae oil ratio of 1:2. However, the possibility of a sex-based difference in the anti-aging effect of the tuna oil and algae oil mixture or gut microbiota variation, has rarely been investigated. In this study, the anti-aging effect of an oil mixture (1:2) in male and female mice was measured, and oil treatment improved the learning and cognition of mice that were damaged by D-gal, increased the activities of anti-oxidative enzymes, and decreased the level of MDA, which acted as a hallmark of oxidative damage to lipids. Male mice showed better anti-aging effects than female mice with a specific oil mixture ratio, and the clinical drug donepezil showed a similar or better effect on aging alleviation than oil treatments in both sexes. On the other hand, the same oil treatment led to different gut microbiota composition alterations in male and female mice. Redundancy analysis (RDA) identified 31 and 30 key operational taxonomic units (OTUs) in the male and female mice, respectively, and only three of these OTUs overlapped. Moreover, the abundance of *Lactobacillus* and several probiotic-like butyric acid producers was higher in male mice than in female mice, whereas the abundance of some inflammation-related genera, such as *Clostridium XlVa*, was lower in male mice. In conclusion, this study indicated the sex-based differences related to the anti-aging effects of tuna oil and algae oil treatment are accompanied by sex-based differences in gut microbiota modulation.

## Introduction

At present, the number of aging people with nervous system defects is increasing. There is growing evidence that aging is mainly associated with cognitive impairment in the absence of neurological conditions and comes with an accelerated risk of neurological diseases, such as Alzheimer’s disease (AD) and Parkinson’s disease, due to complex interactions between the environment, lifestyle and genes. In accordance with the free radical theory of aging proposed by Harman (1956), abnormalities in oxidative metabolism produce excess reactive oxygen species (ROS), which act as signaling molecules instead of as metabolic by-products, leading to reversible or irreversible proteins modification, hampering the repair of damaged nuclei, and causing cell stress responses to age-dependent damage (Tönnies and Trushina, 2017). D-galactose (D-gal) is a reducing sugar that can be converted to galactitol after long-term administration at a high dose, and galactitol increases ROS production. In addition, ROS and advanced glycation end (AGE) products derived from D-galactose can induce inflammation in the brain via the NF-κB signaling pathway (Lu et al., 2010).

Tuna oil and algae oil are rich in omega-3 polyunsaturated fatty acids, and various previous studies indicated that omega-3 polyunsaturated fatty acids supplementation increased excitability of neuronal membrane, level of neurotransmitter, growth of hippocampal neurons, as well as learning acquisition and memory performance (Carrié et al., 2000; Ikemoto et al., 2000; Morris et al., 2003). Docosahexaenoic acid (DHA) and eicosapentaenoic acid (EPA), are important omega-3 polyunsaturated fatty acids, and accumulating evidence indicated that DHA and EPA treatments will inhibit Aβ generation, suppressed apoptosis, down-regulated inflammatory response, improved neurotrophic activity, and ameliorate memory and cognitive function (Che et al., 2018). Until recently, the DHA, EPA and arachidonic acid (AA) are regarded as the key factors for anti-ageing effects. They induced the expression of brain-derived neurotrophic factor, and their derivatives (4-hydroxyhexenal and 4-hydroxynonenal) activated the Nrf2 pathway and protected neurons against oxidative stress (Wu et al., 2004; Chen et al., 2005).

Indeed, the microbiota and its metabolites have also been proposed to be involved in various brain function modulations, such as emotional behaviors, stress-related responsiveness, pain and food intake (Cryan and Dinan, 2012; Thakur et al., 2014; Mathilde et al., 2018). Thus, alterations of the microbiota via various factors of “unhealthy life styles”, such as diet, drug use and stress, may lead to functional and behavioral deterioration. A high-fat diet, proposed as a factor in obesity, is suggested lead to the dysbiosis of gut microbiota and trigger mood disorders, such as anxiety and depression (Bridgewater et al., 2017). A 2-week antibiotics treatment causes gut endocannabinoidome changes, hippocampal neuroglial reorganization and depression in mice (Guida et al., 2018), whereas low-dose antibiotic treatments in early life induce long-term changes in gut microbiota, brain cytokines and behavior (Leclercq et al., 2017). The microbiota-gut-brain axis regulates the bidirectional interactions between the intestine and the central nervous system (CNS). On the one hand, the brain-intestinal axis alters the composition of the gut microbiota through neuroendocrine, immune and humoral mechanisms. On the other hand, the gut microbiota improves the plasticity of abnormal brains, increasing stress hormone secretion via its metabolites, such as γ-aminobutyric acid (Ait-Belgnaoui et al., 2014).

Individual differences in host microbial community structure are caused by environmental and genetic factors (Org et al., 2016). Although sex differences have a significant effect on physiology and behavior, it is difficult to demonstrate sex differences in the composition of the gut microbiota (van Nas et al., 2009). Sex hormones have a certain effect on the formation of gut microbiota, but their mechanisms are unknown. Moreover, there are sex differences related to disease prevalence and symptoms, such as those in autism, drug abuse, depression and AD (Canevelli et al., 2017; Coretti et al., 2017). However, it remains unknown whether the gut microbiota structural modulation that occurs during the alleviation of aging by oil treatment is sex-dependent.

Our previous study indicated that oil treatment can alleviate aging in male mice and is accompanied by structural changes in the gut microbiota (Zhang et al., 2018). Oil supplementation enriched the abundance of* Lactobacillus*, *Bacteroides*, *Coprobacter*, *Tannerella* and *Prevotella* and decreased the abundance of *Falsiporphyromonas*. Redundancy analysis (RDA) identified 83 operational taxonomic units (OTUs) that responded to oil treatment, five of which were significantly related to one or more host aging parameters. In this study, a similar experiment in female mice based on the results of previous male mouse studies was conducted. In addition, sex differences were identified regarding the effect of dietary supplementation with mixed oil (tuna oil and algae oil at a 1:2 ratio as previously described) on aging. Moreover, the relationship between the treatment effect and specific bacterial taxa in aging females and males was explored. Our study indicated that the sex-based differences related to the anti-aging effects of tuna oil and algae oil treatment are accompanied by sex-based differences in gut microbiota modulation.

## Materials and Methods

### Experimental Design

All experimental procedures and animal care accorded with the experimental animal care and use guidelines prepared by the Ningbo University Experimental Animal Center (affiliated with the Zhejiang Laboratory Animal Common Service Platform, Ningbo, China), and all animal programs received the approval of the Ningbo University Laboratory Animal Center under permit number SCXK (ZHE 2014-0001).

In total, 192 6-week-old ICR mice (96 male and 96 female; male mice 24.1 ± 2.6 g; female mice 21.1 ± 1.6 g) were adapted to a standard diet for 2 weeks. After 2 weeks, their baseline body weight was measured, and fecal samples were collected randomly from six mice for microbiome analysis (0 week). Subsequently, the 192 mice were divided into eight treatment groups (12 male and 12 female mice per group, four to a cage). The control mice were fed a standard diet (Laboratory Animal Center of Ningbo University, Ningbo, China) and received an intraperitoneal injection of saline (180 mg·kg^−1^·d^−1^), and the mice in other groups received an intraperitoneal injection of D-gal (180 mg·kg^−1^·d^−1^) for 12 weeks. The control and D-gal groups simultaneously received saline by gavage. The mice in the D-gal+D group received donepezil (1 mg·kg^−1^·d^−1^) by gavage for 12 weeks, and the donepezil was used as a treatment for aging. The remaining five groups were fed 600 mg·kg^−1^·d^−1^ tuna oil (TO600 group), 600 mg·kg^−1^·d^−1^ algae oil (AO600 group), or a mixture of tuna oil and algae oil at a ratio of 1:1, 1:2 or 1:3 (600 mg·kg^−1^·d^−1^, TO300AO300 group, TO200AO400 group and TO150AO450 group, respectively) for 12.

Body weight was measured every week. After 12 weeks of feeding, fecal samples were collected from each cage, immediately placed in liquid nitrogen, and stored at −80°C. The animals were anesthetized by isoflurane (Hui et al., 2017). Blood was collected from mouse eye sockets, and serum was centrifuged at 3000 rpm for 15 min at 4°C and stored at −80°C for biochemical tests. Subsequently, mice were sacrificed by cervical dislocation, and their organs, including the kidney, thymus, brain, heart, spleen, liver and lung, were excised, weighed and immediately stored in liquid nitrogen. The visceral index was calculated as follows: organ weight/body weight (mg/g; Lu et al., 2017).

Our previous study sequenced the fecal samples of male mice in the eight groups (control, D-gal, D-gal+D, TO600, AO600, TO300AO300, TO200AO400 and TO150AO450 groups), and the treatment of tuna oil and algae oil at a ratio of 1:2 showed the best anti-aging effects in male mice (Zhang et al., 2018). Therefore, in female mice, fecal samples in the responding six groups (control, D-gal, D-gal+D, TO600, AO600 and TO200AO400 groups) were sequenced and compared with the data obtained from male mice in the responding groups.

### Morris Water Maze

Mice can learn and remember how to find a hidden platform in the Morris water maze (MWM) test. The MWM test was carried out as previously described (Zhou et al., 2013). The mice were tested in the same order at the same time every day using the same MWM and space environment. Tests were run four times a day for four consecutive days. After each test, the mice were dried under a heat lamp. To clarify spatial memory retention after a period of time, a probe test was performed on day 5. We used water maze software and a tracking system to measure the daily escape latency to the hidden platform, the number of platform crossings in the target quadrant, and swimming speed during the probing test by a camera mounted directly above the MWM.

### Measurement of Brain Biochemical Indices

Glutathione peroxidase (GSH-Px), total anti-oxidant capacity (T-AOC), malondialdehyde (MDA), superoxide dismutase (SOD) and catalase (CAT) in the brain were measured using their corresponding kits (Nanjing Jiancheng Bioengineering Institute, Nanjing, China).

### Total DNA Extraction, PCR and Sequencing

Total DNA containing fecal microbial communities was extracted from the mouse fecal samples as previously described (Yu and Morrison, 2004). The extracted DNA was measured by a Thermo NanoDrop 2000C (Thermo Fisher Scientific, MA, USA).

The PCR primers 341F 5’-CCTACGGGNGGCWGCAG-3’ and 805R 5’-GACTACHVGGGTATCTAATCC-3’ with unique bar code sequences for each sample were designed in the V3 and V4 hypervariable region of the bacterial 16S rRNA gene. The amplification reaction was carried out in a 25 μL volume, which contained 20 ng of template, 12.5 μL of Premix Ex Taq™ Hot Start Version and 0.1 μM primer. Amplification started at 98°C for 30 s, followed by 35 cycles of denaturation at 98°C for 10 s, annealing at 54°C for 30 s, extension at 72°C for 45 s, and a final extension for 10 min. The presence of amplicons was confirmed by gel electrophoresis, and the PCR product was normalized using an AxyPrep™ Mag PCR Standardizer. Sequencing was performed using the MiSeq system constructed by the Illumina Nextera XT index kit from Sangon Biotech Co., Ltd. (Shanghai, China). According to the manufacturer’s instructions, sequencing was performed on the Illumina MiSeq (Illumina, CA, USA) using 2 × 300 bp paired-end sequencing and multiplex sequencing run.

### Data and Statistical Analysis

As previously described (Lu et al., 2017), the raw FASTQ files were filtered and multiplexed using QIIME (version 1.8.0; Caporaso et al., 2010) and PEAR (version 0.9.6; Zhang et al., 2014) as follows: (1) reads with more than 10 bp of overlap were merged, and reads that could not be merged were removed; (2) data belonging to each sample were identified through the barcode sequence; (3) reads were truncated at any position when the average quality score on the 10-bp sliding window was below 20; and (4) reads containing fewer than 200 bp or undetected nucleotides (N) were removed. Chimera sequences were identified and removed using Uchime (version 4.2.40; Edgar et al., 2011). OTUs were clustered using Usearch (version 7.1) at 97% similarity (Edgar, 2010). The most abundant sequences in each OTU were defined as the typical sequence and used for taxonomic classification by RDP Classifier (Wang et al., 2007). The indices of community diversity and community richness were calculated via Mothur (version 1.30.1; Schloss et al., 2009). Principal co-ordinates (PCoA) analysis was completed via Muscle (version 3.8.31; Edgar, 2004), FastTree (2.1.3; Price et al., 2010), and the vegan package in R (version 3.2). Heatmap analysis was obtained via HemI (version 1.0.3.3; Deng et al., 2014).

All the data are shown as the mean ± SEM. The ANOVA test and Tukey’s *post hoc* test (SPSS, version 19.0, Chicago, IL, USA) were used to analyze data with a normal distribution, and the Mann-Whitney test (MATLAB R2012a, Natick, MA, USA) was used to analyze data that did not meet the assumptions of the ANOVA. *P* < 0.05 was defined as the standard criterion for statistical significance. Redundant analytical models were constructed to identify D-gal-specific and/or oil-treated-specific bacterial traits. According to the manufacturer’s instructions, the relative abundance of each OTU was normalized and used to build the RDA model to find OTUs that were different between groups using Canoco for Windows 4.5 (Microcomputer Power, Ithaca, NY, USA). The type of treatment was used as an environmental variable. Spearman correlation coefficients (R) and *P* values were used to calculate the correlation between gut microbiome composition and aging phenotype. Heat map analysis of the R value of the relationship between the OTU abundance and the biochemical index was performed using HemI software. When *P* < 0.05 and false discovery rate <0.25, the correlation between the abundance of the gut microbiota and the aging phenotype was considered significant (Lu et al., 2017).

### Accession Numbers

The sequences of the fecal samples in the eight groups of male mice (control, D-gal, D-gal+D, TO600, AO600, TO300AO300, TO200AO400 and TO150AO450 groups) have been deposited in the NCBI sequence read archive database with the accession number SRP101597. The accession number for the sequences of the fecal samples in the six groups of female mice (control, D-gal, D-gal+D, TO600, AO600 and TO200AO400 groups) is SRP127698.

## Results

### Effects of Donepezil and Oil Treatments on Body Weight and Organ Indices

**Figure 1:**
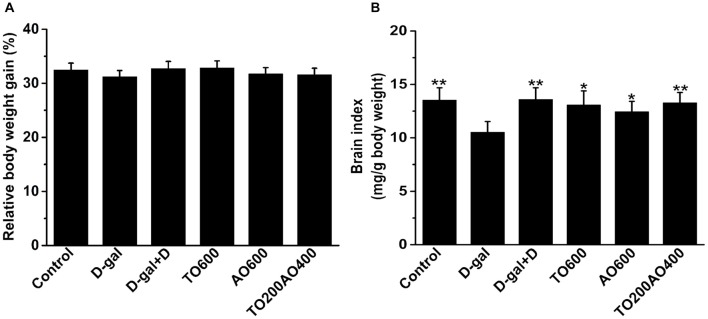
Effects of oil treatment on body weight and brain index in female mice.** (A)** The relative body weight gain of D-galactose (D-gal)-treated mice with oil treatment. **(B)** Changes in the brain index of female mice that received oil treatment. The data are shown as the mean ± SEM, *n* = 12 per group. *P < 0.05, **P < 0.01 vs. D-gal group.

Donepezil and oil treatments showed no effect on body weight gain in female mice (*P* > 0.05; Figure 1A), and the results were the same in male mice (Supplementary Table S1).

In female mice, compared with the D-gal group, donepezil and oil treatments increased the brain index (Figure 1B), kidney index, heart index, spleen index and lung index and reduced the liver index (*P* < 0.05), whereas the thymus index did not significantly change after donepezil or oil treatments (Supplementary Figure S1).

### Effects of Supplemental Donepezil and Oil Mixture on MWM Test

**Figure 2:**
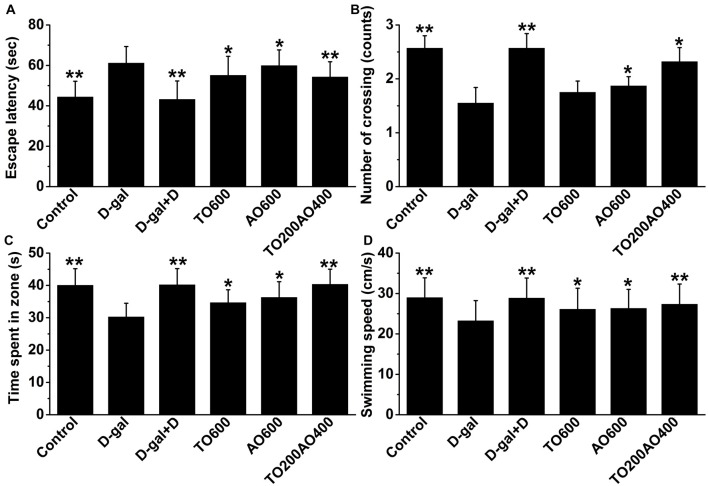
The effects of oil treatment on memory and spatial learning in D-gal-treated female mice. **(A)** Escape latency to the hidden platform on the 5th day. **(B)** The exact number of platform crossings on the 5th day. **(C)** A comparison of time spent in the target quadrant on the 5th day. **(D)** The swimming speeds on the 5th day. The data are shown as the mean ± SEM, *n* = 12 per group. *P < 0.05, **P < 0.01 vs. D-gal group.

As shown in Figure 2, the escape latency of the D-gal group significantly increased compared with that of the control group, whereas the number of times through the target quadrant, target quadrant residence time and swimming speed were significantly reduced (*P* < 0.01) in female mice, indicating that D-gal-treatment inflicted a cognitive impairment. After treatment with donepezil or an oil mixture, the number of target quadrant crossings, target quadrant residence time and swimming speed increased, whereas the escape latency decreased (*P* < 0.05). The results suggest that both donepezil and oil treatments improved spatial memory and learning ability in the D-gal treatment group, whereas the donepezil treatment showed more similar MWM test results to the control group than the oil treatments. In addition, among the oil treatment groups, the TO200AO400 treatment showed the best anti-aging effect in females, which was similar to what was previously reported in male mice. Compared with the D-gal group, the escape latency (11.24% vs. 11.47%), number of times through the platform (49.68% vs. 83.21%), target quadrant time (25.43% vs. 25.79%), and swimming speed (17.69% vs. 17.68%) improved for the female vs. male TO200AO400 group (Supplementary Figure S2). In conclusion, in the MWM test, the oil treatment showed a better anti-aging effect on male mice.

### Donepezil and Oil Supplementation Reduces Oxidative Damage in D-gal-Induced-Aging Mice

**Figure 3:**
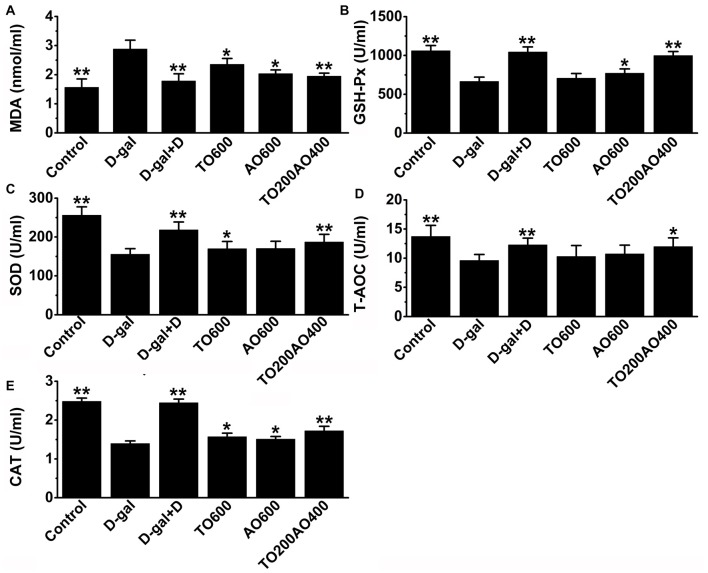
Effects of D-gal, donepezil and oil treatments on malondialdehyde (MDA; **A**), Glutathione peroxidase (GSH-Px; **B**), superoxide dismutase (SOD; **C**), total anti-oxidant capacity (T-AOC; **D**) and catalase (CAT; **E**) in the brain. The data are shown as the mean ± SEM, *n* = 12 per group. *P < 0.05, **P < 0.01 vs. D-gal group.

The oxidative and anti-oxidative systems are balanced in healthy individuals. However, if there is an increase in oxidative damage that cannot be overcome by anti-oxidative system, lipids in the cell are damaged, and MDA acts as a hallmark of oxidative damage in lipids. In addition, the anti-oxidative system consists of reducing substances and anti-oxidative enzymes, such as CAT, GSH-Px and SOD. In female mice, the activities of T-AOC, GSH-Px, SOD and CAT in the brain were significantly decreased (*P* < 0.01) in D-gal mice compared with the control group, whereas MDA levels were significantly increased (*P* < 0.01; Figure 3). After donepezil or oil treatments, the activities of T-AOC, GSH-Px, SOD and CAT increased, and the MDA level decreased, and donepezil treatment restored these indices to values closest to the control group compared with the other oil treatments (Figure 3).

Similar to the results obtained in the MWM test, the TO200AO400 treatment showed the best anti-aging effect in females. Compared with the D-gal group, the MDA (62.23% vs. 64.88%), GSH-Px (150.88% vs. 151.02%), SOD (119.74% vs. 122.17%), T-AOC (110.93% vs. 112.08%) and CAT (112.89% vs. 114.53%) improved for the female vs. male TO200AO400 group (Supplementary Table S2, Supplementary Figure S3). In conclusion, in the anti-oxidative indices, the oil treatment showed a better anti-aging effect on male mice.

### Donepezil and Oil Supplementation Lead to Changes in Gut Microbiota Structure

**Figure 4:**
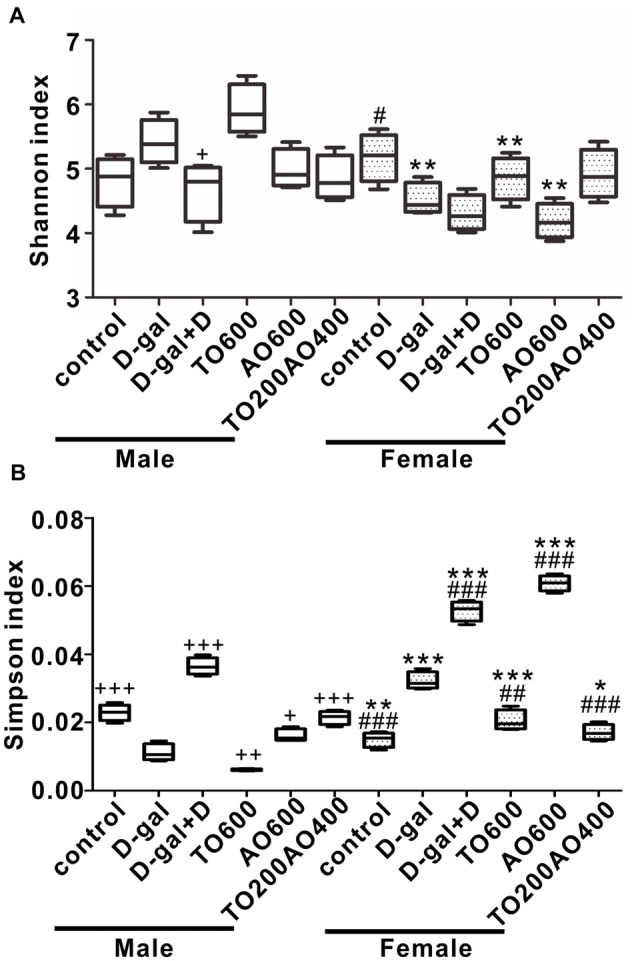
The alpha diversity of the gut microbiota in the different groups of female and male mice.** (A)** Shannon index; **(B)** Simpson index. ^+^*P* < 0.05, ^++^*P* < 0.01 and ^+++^*P* < 0.001 vs. D-gal group in the male. ^#^*P* < 0.05, ^##^*P* < 0.01 and ^###^*P* < 0.001 vs. D-gal group in the female. *P < 0.05, **P < 0.01 and ***P < 0.001, corresponding to the treatment group in male vs. female mice, and *n* = 3 per group.

After 12 weeks of feeding, we sequenced fecal samples to elucidate the contribution of donepezil or oil treatments on the gut microbiota structure. In male mice, the Shannon index increased after D-gal treatment (*P* > 0.05), and the subsequent donepezil (*P* < 0.05), AO600 or TO200AO400 (*P* > 0.05) treatments decreased the Shannon index, whereas the TO600 treatment further increased it (*P* > 0.05). In female mice, the Shannon index decreased after D-gal treatment (*P* < 0.05), increased after TO600 and TO200AO400 treatments (*P* > 0.05) and further decreased by donepezil and AO600 treatments (*P* > 0.05; Figure 4A). In total, opposite trends were observed in the Simpson index in male and female mice (Figure 4B). In addition, the Chao1 index and ACE index showed similar patterns to the Shannon index (Supplementary Figure S4).

**Figure 5:**
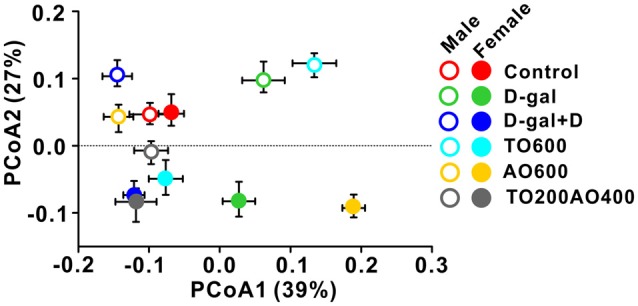
Weighted UniFrac Principal co-ordinates (PCoA) analysis of the gut microbiota in male and female mice with different treatments.

The overall structure of the gut microbiota in six groups of mice was analyzed by weighted UniFrac PCoA, and the structure of the gut microbiota in male mice was independent of the structure in female mice, except for in the control. Similar gut microbiota structures were observed in the control groups (red triangle vs. red circle), whereas the same treatment types led to different gut microbiota structures between male and female mice (triangle vs. circle for same color group; Figure 5).

### Response to Oil-Treated Gut Microbiota Shifts in D-gal-Induced-Aging Mice

Donepezil is a clinical drug inhibited the activity of acetylcholinesterase, and thereby enhance cognitive function. In this study, it is used as the positive control, and showed effects of anti-aging and gut microbiota modulation. However, in subsequent gut microbiota data analysis, we excluded this group based on the following considerations. On the one hand, previous study indicated that it will cause gastrointestinal side effects, induce the gastrointestinal into a sub-healthy status (Mimica and Presecki, 2009), and we proposed the information obtained from this group might not completely link to the anti-aging effect, somehow to the side-effects. On the other hand, donepezil is a pyridine derivative rather than a polyunsaturated fatty acid, and different mechanisms might exist in gut microbiota modulation with totally different compounds. Thus, if we take it into consideration together with oil treatment, it might be misleading.

In female and male mice, the relative abundances of *Bacteroidetes* and *Firmicutes* decreased in the D-gal group compared with the control group, and the relative abundance of *Proteobacteria* increased. A wide range of changes in gut microbiota structure were observed at the phylum level. Twelve weeks of treatment with TO200AO400 reversed the changes in the gut microbiota community structure (Supplementary Figure S5).

The proportions of *Roseburia*, *Oscillibacter* and *Parabacteroides* were higher in female mice than in the male mice in all groups, whereas the proportions of *Saccharibacteria*, *Rikenella* and *Intestinimonas* were higher in male mice than in female mice. After D-gal treatment in female and male mice, the proportion of *Alistipes* increased, and the proportions of *Lactobacillus*, *Clostridium XlVa*, *Desulfovibrio*, *Clostridium IV*, *Macellibacteroides* and *Lachnospiracea incertae sedis* decreased (Supplementary Table S3). After oil treatment, the proportions of *Barnesiella*, *Bacteroides*, *Coprobacter*, *Tannerella* and *Clostridium XlVa* reversed to normal levels in female and male mice. The proportion of *Rikenella* increased in both sexes, and the proportions of *Macellibacteroides* and *Lachnospiracea incertae sedis* decreased in both sexes (Supplementary Table S3).

### Key Phylotypes Responsive to Oil Treatment in D-gal-Induced-Aging Mice

**Figure 6:**
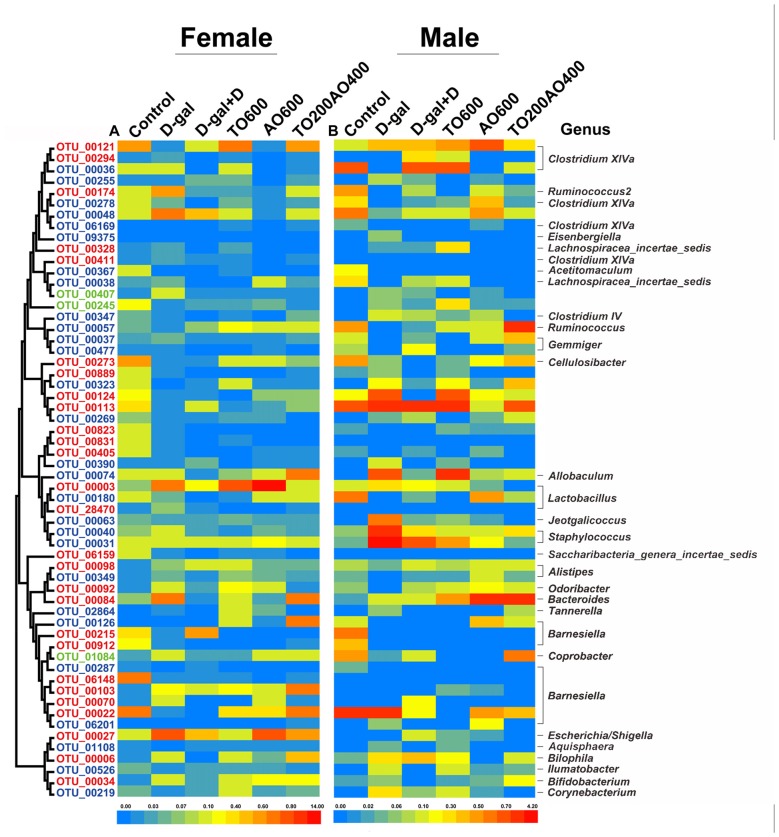
The abundance of 58 operational taxonomic units (OTUs) was altered by D-gal and oil treatment based on redundancy analysis (RDA). **(A)** The heatmap for female mice. **(B)** The heatmap for male mice. The OTUs are sorted by phylogenetic position. The OTUs with higher abundance in females are labeled in red, whereas the OTUs with higher abundance in males are labeled in blue. Green represents the OTUs that were found in both sexes. The color of the heatmap represents the normalized relative abundance of the 58 OTUs. The taxonomy (genus) of the OTUs is at the right. The color of the squares represents the average abundance of the OTUs, and *n* = 3 per group.

Fifty-eight key OTUs in response to tuna oil and algae oil treatment were identified by RDA (Figure 6, Supplementary Table S4 and Supplementary Table S5). Among the 58 OTUs, 30 OTUs and 31 OTUs were respectively found in female and male mice, and three OTUs were found in both female and male, namely, OTU00245, OTU01048 and OTU00407. The first two OTUs belonged to unclassified *Firmicutes*, and the remaining OTU belonged to *Barnesiella*. In female and male mice, 10 and 19 OTUs were respectively reversed by oil treatment, and four OTUs were found in both female and male mice; these OTUs belonged to *Clostridium XlVa* (*n* = 1), *Ruminococcus2* (*n* = 1) and unclassified *Firmicutes* (*n* = 2), all of which are in the phylum* Firmicutes*.

**Figure 7:**
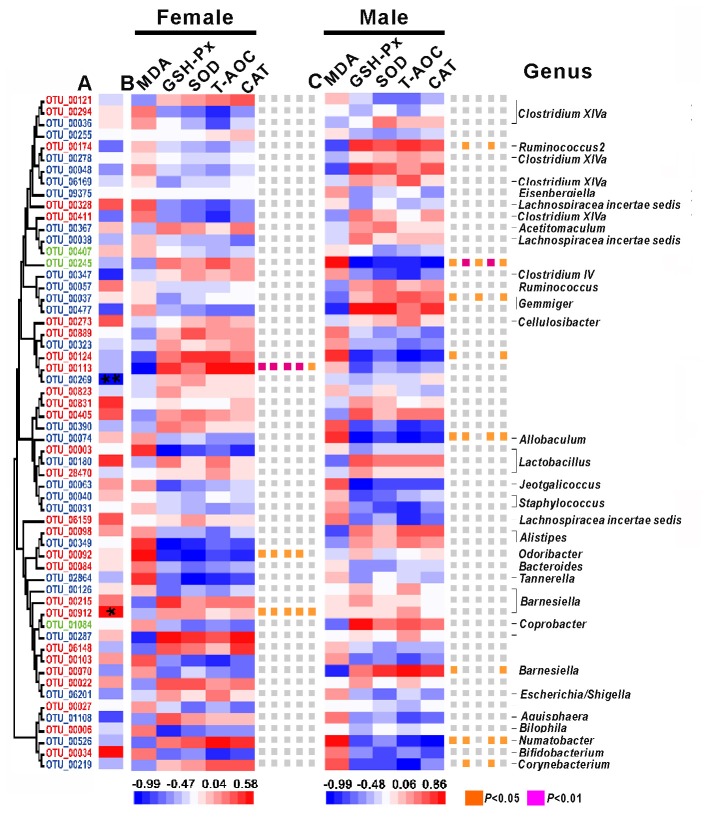
The correlations between phenotypes and OTU abundance was obtained via SPSS in Spearman mode. **(A)** The correlation between females and males. Significant associations are marked with an asterisk *, *P < 0.05 and **P < 0.01. **(B)** The correlations between phenotypes and OTU abundance in females. **(C)** The correlations between phenotypes and OTU abundance in males. The OTUs are ordered by their phylogenetic positions, and the classification of each OUT’s genus is at the bottom.

Among the 58 key OTUs, two OTUs showed significant differences in female and male mice, namely, OTU00269 (unclassified *Firmicutes*; *P* < 0.01) and OTU00912 (*Barnesiella*; *P* < 0.05). In female mice, three OTUs showed a significant correlation with phenotypes, namely, OTU00113, OTU00092 and OTU00912, belonging to unclassified *Firmicutes*, *Odoribacter* and *Barnesiella*, respectively. In the male mice, eight OTUs showed significant correlations with phenotypes, namely, OTU00174, OTU00245, OTU00124, OTU00037, OTU00074, OTU00070, OTU00526 and OTU00219, belonging to *Ruminococcus2*, unclassified *Firmicutes* (*n* = 2), *Gemmiger*, *Allobaculum*, *Barnesiella*, *Ilumatobacter* and *Corynebacterium*, respectively (Figure 7). None of the same key OTUs were found in both female and male mice. These results demonstrated sex-based differences in the gut microbiota during D-gal-induced aging progression and alleviation.

## Discussion

Although dietary tuna oil and algae oil supplementation has beneficial metabolic effects (Doughman et al., 2013; Perez-Pardo et al., 2017), little information is available concerning its mechanism of action, especially in terms of sex differences. In this study, we found that concomitant with the alleviation of D-gal-induced aging, altered microbial composition induced by tuna oil and algae oil was observed, and a better anti-aging effect was obtained in male mice than in female mice.

D-gal induces oxidative damage, such as high levels of ROS, and leads to neurotoxicity in mice, and the anti-oxidative systems (CAT, GSH-Px and SOD, etc.) are responsible for reducing the damage caused by oxidative stress. In this study, the D-gal-induced damage increased MDA (hallmark of oxidative damage in lipids) and reduced anti-oxidative enzyme activities, indicating the imbalance between oxidative and anti-oxidative systems in the D-gal group. Subsequently, oil treatment re-balanced the oxidative and anti-oxidative systems with decreased MDA levels and enhanced CAT, GSH-Px and SOD. In addition, omega-3 polyunsaturated fatty acids, especially DHA and EPA, play vital roles in alleviating ROS-induced aging. On the one hand, DHA and AA are converted to 4-hydroxyhexenal and 4-hydroxynonenal, respectively, and these derivatives protect neurons against oxidative stress by producing heme oxygenase-1 and activating the Nrf2 pathway (Chen et al., 2005, 2006). On the other hand, DHA induced brain-derived neurotrophic factor, which is involved in synaptic transmission regulation and cognitive function improvement (Wu et al., 2004).

D-gal injection and oil administration showed marked effects on cognitive behaviors, and the effects are proposed to be sex-based. In both male and female mice, D-gal injection induced cognitive impairment, and the subsequent oil treatment showed a positive effect on behavior. Moreover, the results of MWM indicated that oil treatments had a better anti-aging effect on male mice than on female mice. Previous studies suggested that the aging process has sex differences, and male mice seem to show greater advantages in this regard. Recent estimates suggest that almost two-thirds of people diagnosed with AD are women (Canevelli et al., 2017) and that women exhibit more robust progress in mild cognitive dysfunction (Lin et al., 2015) and higher rates of severe clinical dementia (Barnes et al., 2005). However, the results obtained from observational studies are controversial: some studies show a higher cognitive efficacy of new cholinesterase inhibitors in female AD patients, whereas other reports indicate that men are more responsive to anti-dementia therapy or that there is no significant sex difference (Haywood and Mukaetova-Ladinska, 2006; Gallucci et al., 2016; Canevelli et al., 2017). Some studies have proposed differences in educational level, life expectancy, sex hormones, genetics and cognitive detection bias to explain this sex difference (Mielke et al., 2014; Canevelli et al., 2017).

In this study, identical dietary supplementation led to different gut microbiota structures in male and female mice, and the male structures were independent of the structures in females, except for the controls (Figure 5). Studies have shown that male mice show significant differences in their gut microbiota after DHA introduction, whereas no significant differences were observed in female mice (Davis et al., 2017). In contrast, the addition of n-3 polyunsaturated fatty acids significantly altered the gut microbiota in C57BL/6 female mice (Ghosh et al., 2013). The above data suggest that future explorations of gut microbiota dietary induction should take sex into consideration as an important variable.

Gastrointestinal-rich gram-positive facultative anaerobic bacteria or *Lactobacillus acidophilus* and other Bifidobacterium species metabolize glutamate to produce gamma-aminobutyric acid, which is the major inhibitory neurotransmitter in the CNS. Functional disorders in gamma-aminobutyric acid signaling pathways are associated with anxiety, depression, synaptic defects and cognitive disorders (Bhattacharjee and Lukiw, 2013). Liang et al. (2015) found that probiotic *Lactobacillus helveticus* NS8 significantly improved the cognitive impairment induced by chronic constrained stress in rats. In this study, *Lactobacillus* was enriched by TO200AO400 treatment, and the proportion of *Lactobacillus* was higher in male mice than in female mice (Supplementary Table S3). *Bacteroides* mainly contains species that are used for carbohydrate and protein fermentation, and there is growing evidence that low calorie diets can delay brain aging (Martin et al., 2006). *Bacteroides* and *Parabacteroides* are the producers of propionic acid, and intraperitoneal injection of propionic acid induces pathological changes in autism spectrum disorders (Coretti et al., 2017). In this study, the proportions of *Bacteroides* and *Parabacteroides* decreased in the D-gal-induced aging mice regardless of sex, and after TO200AO400 treatment, their abundances increased. The intestines of mice fed n-6 polyunsaturated fatty acids contained some *Enterobacteriaceae* and *Clostridium*, which induce inflammation (Ghosh et al., 2013), and the proportion of *Clostridium XlVa* was higher in female mice than in male mice after oil treatment (Supplementary Table S3). One of the focuses of anti-aging drugs is that cross-feeding interactions occur between intestinal bifidobacteria and colonic butyrate-producing bacteria, such as *Faecalibacterium praus-nitzii* and *Roseburia*. These kinds of interactions may be beneficial for the coexistence of bifidobacteria and bacteria that produce butyrate in the human colon (Audrey et al., 2016). In addition, butyric acid is thought to play an important role in aging and age-related diseases because it modulates epigenetic processes by inhibiting histone deacetylase activity (Vaiserman et al., 2017). In our study, the proportion of *Roseburia* was higher in female mice than in male mice in the D-gal group, and the ratio of *Roseburia* decreased in female mice after oil treatment. However, the proportion was restored in male mice (Supplementary Tables S4, S5).

Four OTUs showed restored abundance after oil treatment in both the female and male mice, two of which belonged to unclassified *Firmicutes*, and the others were *Clostridium XlVa* and *Ruminococcus2*. Significantly reduced *Clostridium* bacteria in the gut microbiota is widely observed in high-fat diet treatment and type 2 diabetes mellitus patients (Larsen et al., 2010; Naseer et al., 2014), and type 2 diabetes is a known risk factor for AD pathogenesis (Ahtiluoto et al., 2010). On the other hand, *Ruminococcus* is a key cellulase degrader in the large intestine, cecum or rumen, and it is elevated in patients with neurobehavioral disorders (Wang et al., 2013; Li et al., 2017).

The experiment results in male and female mice all supported that donepezil treatment showed significant beneficial effects in D-gal-induced aging. Donepezil is a clinical drug inhibited the activity of acetylcholinesterase and butyrylcholinesterase, and thereby enhance cognitive function (Agunloye and Oboh, 2017). However, acetylcholinesterase inhibitor (AChE) cause gastrointestinal side effects, as well as nausea, vomiting, weight, loss and sleep disturbances (Mimica and Presecki, 2009), whereas few side effect case of tuna oil and algae oil with recommended dose was reported. On the other hand, the donepezil treatment decreased the abundance of several OTUs which belong to genera *Bifidobacterium*, *Lactobacillus* and *Lachnospiracea*, whereas TO200AO400 treatment increased the responding abundance. All these three genera are short-chain fatty acid (SCFA) producers in the intestinal tract, and various studies indicated that the gut microbiota benefits humans via SCFA production, and deficiency in SCFA production is associated with diseases (Zhao et al., 2018). Therefore, we proposed that, compared with the omega-3 polyunsaturated fatty acids, the donepezil treatment might lead to the gut microbiota dysbiosis, and subsequently increased the risk in other diseases.

In conclusion, we found that the anti-aging effects and gut microbiota modulation of the oil mixture treatment were sex dependent and that a specific ratio of oil mixture treatment (1:2 ratio of tuna oil to algae oil) showed better anti-aging effects in male mice than in female mice. Although translating these findings into a clinical population remains a major challenge, it is important to consider sex differences in the clinical presentations of aging and the need for sex-dependent treatments.

## Author Contributions

CL and XS designed the study. ZW, HZ, YanyanL, JH and CC performed the experiments. JZ, LC, YeL, TS, DZ and XS provided reagents and materials. CL, ZW and HZ analyzed the data. CL, ZW, HZ and XS wrote the main manuscript text and prepared figures. All authors reviewed the manuscript.

## Conflict of Interest Statement

The authors declare that the research was conducted in the absence of any commercial or financial relationships that could be construed as a potential conflict of interest.

## Supplementary Material

FIGURE S1 Effects of D-gal, donepezil and oil treatments on the viscera indices. All data are represented as the mean ± SEM, *n* = 12 per group. **P* < 0.05, ***P* < 0.01 vs. D-gal group.

FIGURE S2 Effects of D-gal, donepezil and oil treatments on the morris water maze (MWM) test results in males and females. All data are represented as the mean ± SEM, *n* = 12 per group. **P* < 0.05, ***P* < 0.01 vs. D-gal group.

FIGURE S3 Effects of D-gal, donepezil and oil treatments on MDA, GSH-Px, SOD, CAT and T-AOC in the brain in males and females. All data are represented as the mean ± SEM, *n* = 12 per group. **P* < 0.05, ***P* < 0.01 vs. D-gal group.

FIGURE S4 The alpha diversity of the gut microbiota in the different groups of female and male mice. **(A)** Chao1 index; **(B)** ACE index. ^+^*P* < 0.05 and ^++^*P* < 0.01 vs. D-gal group in male mice. ^#^*P* < 0.05, ^##^*P* < 0.01 and ^###^*P* < 0.001 vs. D-gal group in female mice. **P* < 0.05, ***P* < 0.01 and ****P* < 0.001 in corresponding treatment group for males vs. females and *n* = 3 per group.

FIGURE S5 Relative abundances of bacteria at the phylum level in male and female mice.

TABLE S1 The relative body weight gain of D-gal-treated mice with oil supplementation. The data are expressed as the means ± SEM, *n* = 12.

TABLE S2 Effect of tuna oil, algae oil and mixed treatment on the Glutathione peroxidase (GSH-Px), Superoxide dismutase (SOD), Catalase (CAT) and Total antioxidant capability (T-AOC) activities in the brain of the D-gal-induced aging mice. All data are represented as means ± SEM, *n* = 12 per group. **P* < 0.05, ***P* < 0.01 vs. D-gal group.

TABLE S3 The abundance of genera with sex variations in control, D-gal and TO200AO400 groups in male and female mice. The data are expressed as the mean, *n* = 3 per group.

TABLE S4 Taxonomic assignments of the 58 altered OTUs responding to oil treatments.

TABLE S5 Relative abundance of the 58 altered OTUs responding to tuna oil, algae oil and mixed treatment identified by RDA.
