# Supplementary figures and images for: Sex-Based Differences in Gut Microbiota Composition in Response to Tuna Oil and Algae Oil Supplementation in a D-galactose-Induced Aging Mouse Model
Source: Front Aging Neurosci. 2018 Jun 26;10:187. doi: 10.3389/fnagi.2018.00187 (PMC6028736; doi:10.3389/fnagi.2018.00187)

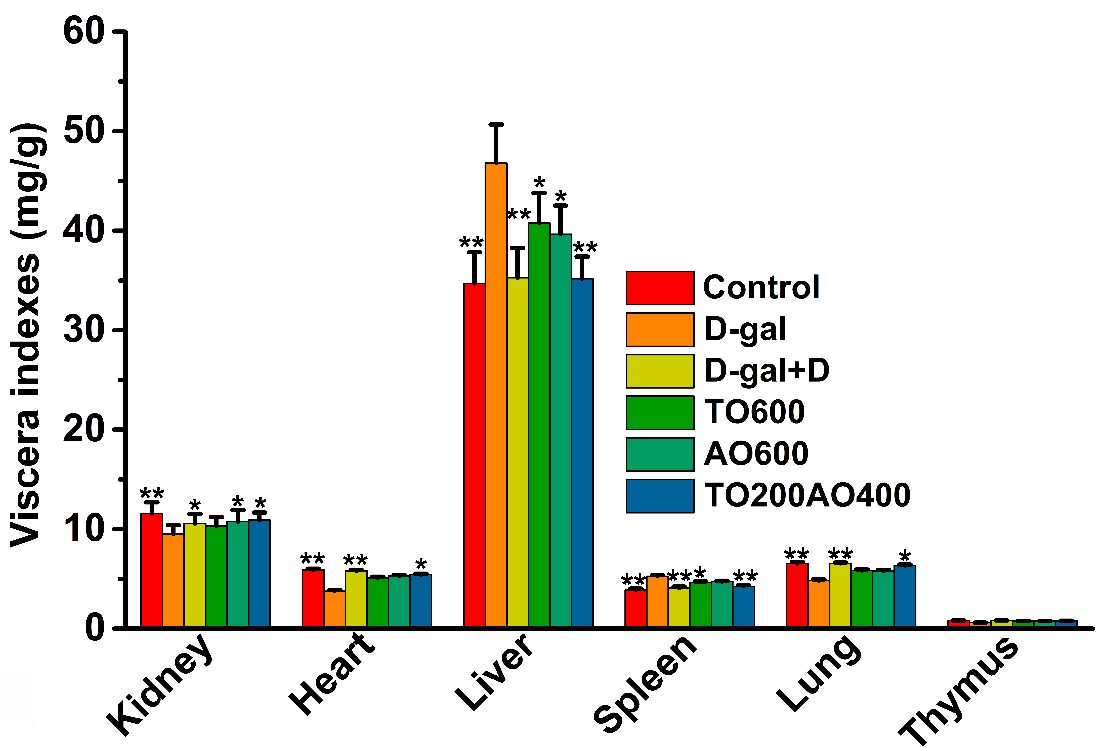

Supplement: FIGURE S1 — Effects of D-gal, donepezil and oil treatments on the viscera indices. All data are represented as the mean ± SEM, n = 12 per group. *P < 0.05, **P < 0.01 vs. D-gal group. [file Image_1.TIF]

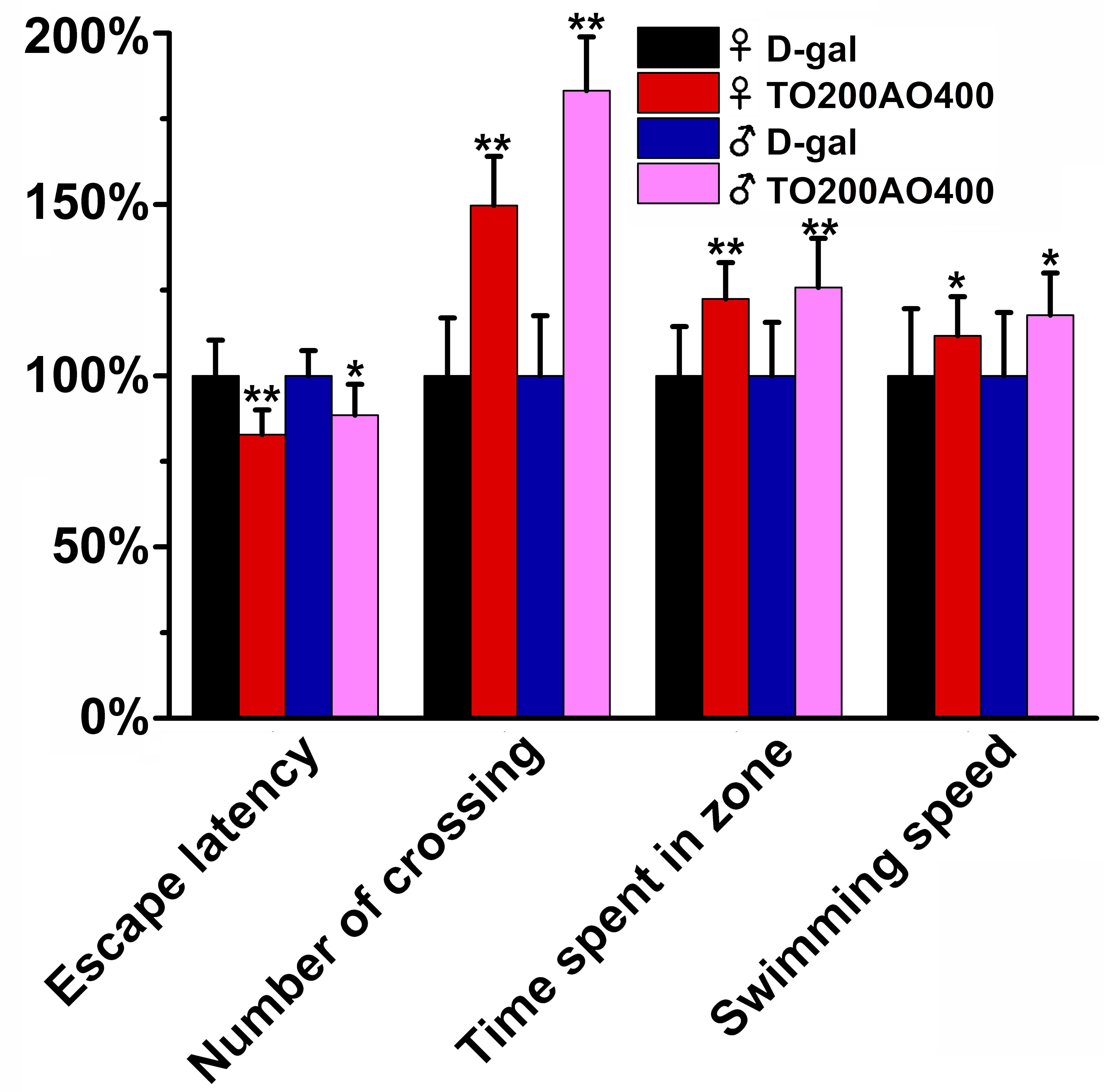

Supplement: FIGURE S2 — Effects of D-gal, donepezil and oil treatments on the morris water maze (MWM) test results in males and females. All data are represented as the mean ± SEM, n = 12 per group. *P < 0.05, **P < 0.01 vs. D-gal group. [file Image_2.TIF]

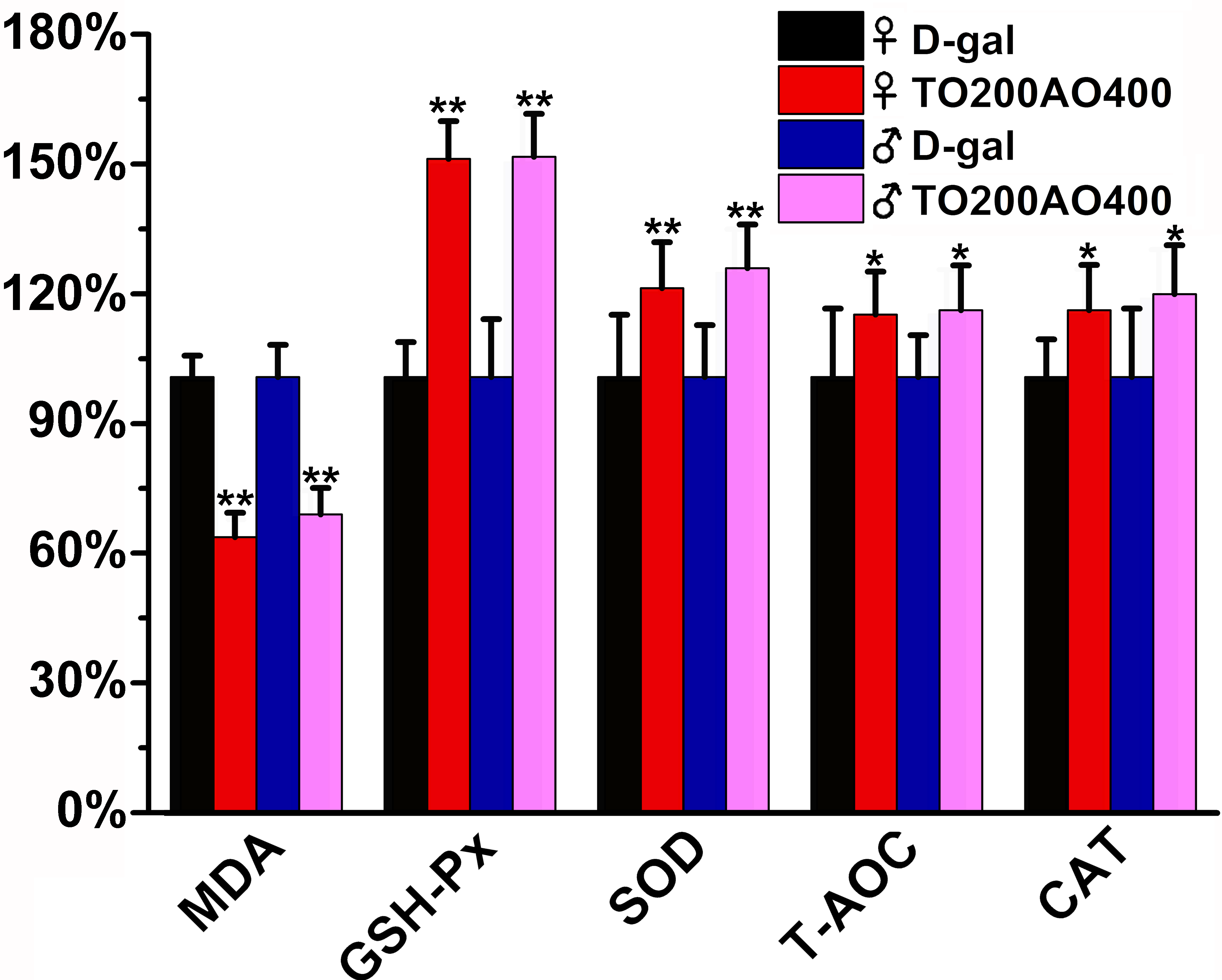

Supplement: FIGURE S3 — Effects of D-gal, donepezil and oil treatments on MDA, GSH-Px, SOD, CAT and T-AOC in the brain in males and females. All data are represented as the mean ± SEM, n = 12 per group. *P < 0.05, **P < 0.01 vs. D-gal group. [file Image_3.TIF]

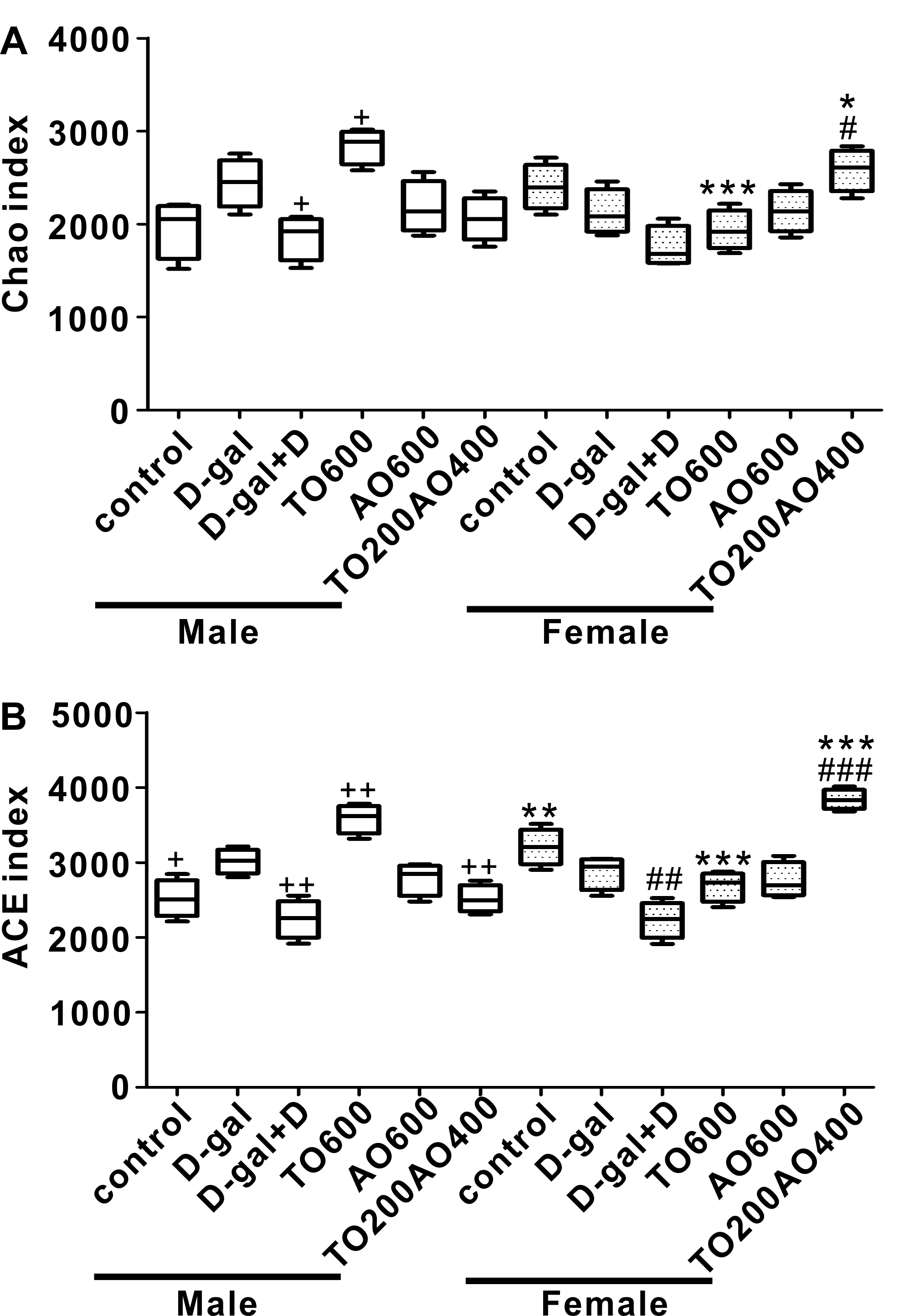

Supplement: FIGURE S4 — The alpha diversity of the gut microbiota in the different groups of female and male mice. (A) Chao1 index; (B) ACE index. +P < 0.05 and ++P < 0.01 vs. D-gal group in male mice. #P < 0.05, ##P < 0.01 and ###P < 0.001 vs. D-gal group in female mice. *P < 0.05, **P < 0.01 and ***P < 0.001 in corresponding treatment group for males vs. females and n = 3 per group. [file Image_4.TIF]

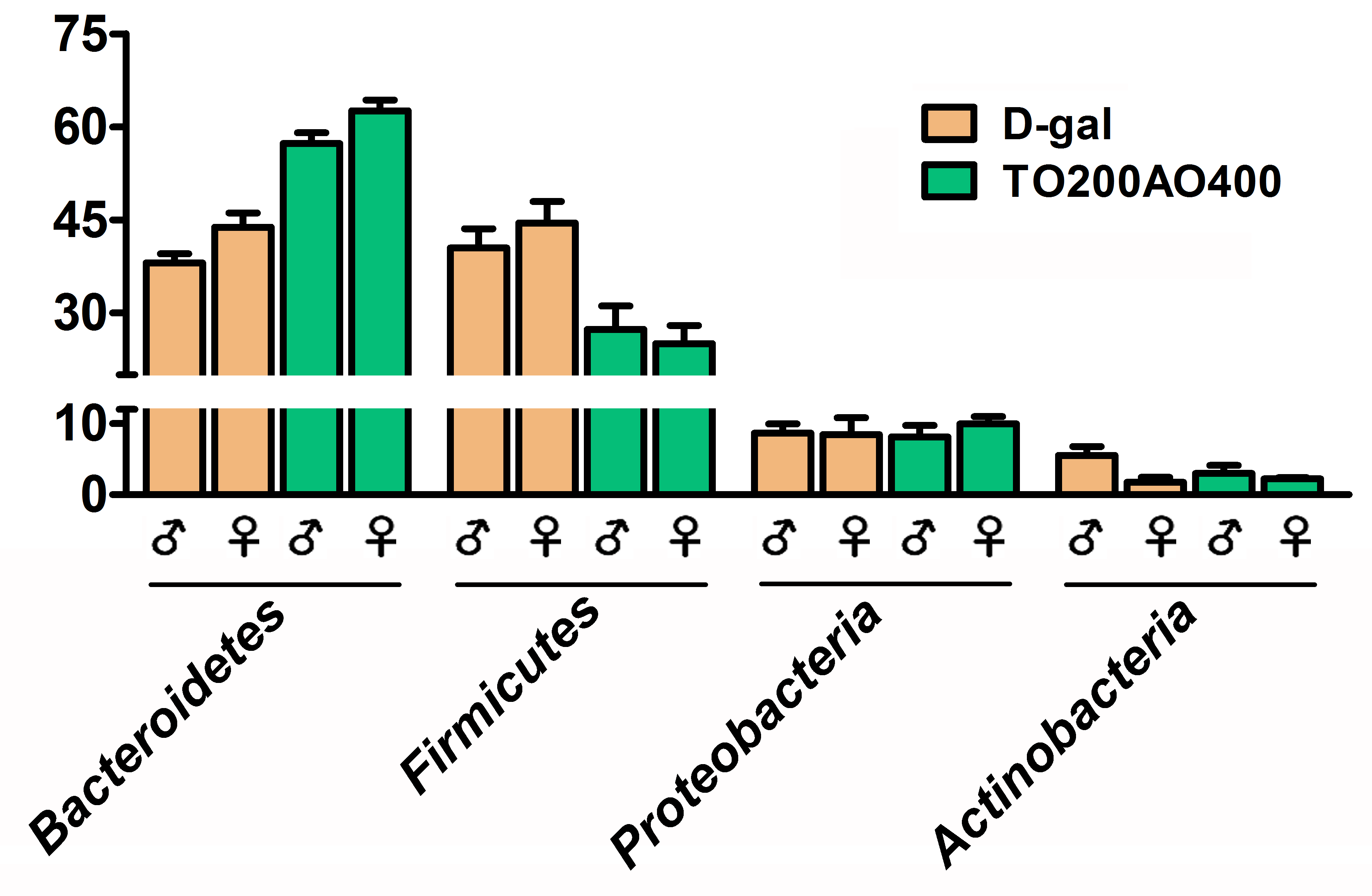

Supplement: FIGURE S5 — Relative abundances of bacteria at the phylum level in male and female mice. [file Image_5.TIF]
