# Supplementary material for: Sex-Based Differences in Gut Microbiota Composition in Response to Tuna Oil and Algae Oil Supplementation in a D-galactose-Induced Aging Mouse Model
Source: Front Aging Neurosci. 2018 Jun 26;10:187. doi: 10.3389/fnagi.2018.00187 (PMC6028736; doi:10.3389/fnagi.2018.00187)
Supplement: TABLE S1 — The relative body weight gain of D-gal-treated mice with oil supplementation. The data are expressed as the means ± SEM, n = 12. [file Table_1.pdf]

Supplementary Table S1. The relative body weight gain of D-gal-treated mice with oil supplementation. The data are expressed as the means ± SEM, n=12.

	Control (g)	D-gal (g)	D-gal+D (g)	TO600 (g)	AO600 (g)	TO200AO400 (g)
female	32.47±1.25	31.22±1.15	32.74±1.30	32.86±1.29	31.75±1.14	31.60±1.17
male	34.47±1.46	30.22±1.28	33.74±1.50	34.86±1.97	31.75±1.37	31.60±1.81
