# Supplementary material for: Sex-Based Differences in Gut Microbiota Composition in Response to Tuna Oil and Algae Oil Supplementation in a D-galactose-Induced Aging Mouse Model
Source: Front Aging Neurosci. 2018 Jun 26;10:187. doi: 10.3389/fnagi.2018.00187 (PMC6028736; doi:10.3389/fnagi.2018.00187)
Supplement: TABLE S2 — Effect of tuna oil, algae oil and mixed treatment on the Glutathione peroxidase (GSH-Px), Superoxide dismutase (SOD), Catalase (CAT) and Total antioxidant capability (T-AOC) activities in the brain of the D-gal-induced aging mice. All data are represented as means ± SEM, n = 12 per group. *P < 0.05, **P < 0.01 vs. D-gal group. [file Table_2.pdf]

Supplementary Table S2. Effect of tuna oil, algae oil and mixed treatment on the GSH-Px (Glutathione peroxidase), SOD (Superoxide dismutase), CAT (Catalase) and T-AOC (Total antioxidant capability) activities in the brain of the D-gal-induced aging mice. All data are represented as means ± SEM, n = 12 per group. *P<0.05, **P<0.01 *vs* D-gal group.

	Group	GSH-Px	SOD	T-AOC	CAT
female	Control	1061.103±66.547**	256.318±21.367**	12.743±1.879**	1.783±0.085**
	D-gal	665.985±54.369	155.585±14.235	9.615±1.013	1.396±0.066
	D-gal + D	1045.204±64.258**	218.489±20.214**	12.294±1.146**	1.447±0.093**
	TO600	707.882±59.361	173.772±18.745*	10.793±1.875	1.572±0.091*
	AO600	772.073±54.216*	170.542±18.312	10.243±1.487	1.511±0.067*
	TO200AO400	998.809±51.361**	187.226±19.547**	10.988±1.487*	1.725±0.113**
male	Control	1135.221±64.523**	283.732±19.245**	14.261±1.627**	1.913±0.097**
	D-gal	651.813±54.217	161.453±17.456	10.512±1.234	1.497±0.084
	D-gal + D	1103.647±49.578**	225.723±18.213**	12.523±1.026**	1.815±0.099**
	TO600	713.673±60.247*	185.306±14.278	10.521±1.548	1.559±0.038*
	AO600	817.355±59.355**	181.954±17.569	10.861±1.355**	1.632±0.085**
	TO200AO400	980.642±49.987**	201.707±20.498*	12.120±1.874**	1.781±0.081**
