# Supplementary material for: Sex-Based Differences in Gut Microbiota Composition in Response to Tuna Oil and Algae Oil Supplementation in a D-galactose-Induced Aging Mouse Model
Source: Front Aging Neurosci. 2018 Jun 26;10:187. doi: 10.3389/fnagi.2018.00187 (PMC6028736; doi:10.3389/fnagi.2018.00187)
Supplement: TABLE S3 — The abundance of genera with sex variations in control, D-gal and TO200AO400 groups in male and female mice. The data are expressed as the mean, n = 3 per group. [file Table_3.PDF]

Supplementary Table S3. The abundance of genera with sex variations in control, D-gal and TO200AO400 groups in male and female mice. The data are expressed as the mean.

	**Female**			**Male**		
	**Control (%)**	**D-gal (%)**	**TO200AO400 (%)**	**Control (%)**	**D-gal (%)**	**TO200AO400 (%)**
Barnesiella	36.58	27.13	45.24	42.2	25.19	37.29
Bacteroides	2.55	1.79	6.04	2.21	1.91	3.14
Coprobacter	4.17	2.5	3.65	3.07	2.53	4.08
Tannerella	1.86	1.81	2.32	1.79	0.89	2.46
Oscillibacter	0.32	0.23	0.26	0.25	0.17	0.2
Cellulosibacter	1.5	0.16	0.36	0.88	0.32	0.76
Saccharibacteria_genera_incertae_sedis	0.17	0.02	0.05	0.37	0.21	0.24
Rikenella	0.51	0.63	0.93	0.72	0.91	1.16
Acetobacteroides	0.62	0.93	0.44	0.71	0.41	0.41
Citrobacter	0.7	0.26	0.27	0.31	0.78	0.54
Bacillariophyta	0.37	0.18	0.21	0.27	1.17	0.38
Alistipes	0.43	1.12	0.63	0.7	0.73	1.55
Lactobacillus	11.93	10.17	9.64	9.07	8.48	11.94
Clostridium XlVa	2.78	1.4	2.37	4.06	3.51	1.32
Desulfovibrio	2.47	0.18	2.55	5.38	1.58	0.16
Clostridium IV	3.2	1.13	0.76	2.82	1.21	1.22
Macellibacteroides	1.23	0.89	0.65	0.89	0.8	0.9
Lachnospiracea_incertae_sedis	0.99	0.24	0.19	0.59	0.48	0.93
Exiguobacterium	1.02	0.52	0.49	0.47	1.12	0.78
Intestinimonas	0.36	0.17	0.14	0.51	1.54	0.26

Parabacteroides	1.77	1.64	0.52	0.27	0.63	0.41
Roseburia	0.22	0.21	0.03	0.09	0.17	0.03
