# Supplementary material for: Sex-Based Differences in Gut Microbiota Composition in Response to Tuna Oil and Algae Oil Supplementation in a D-galactose-Induced Aging Mouse Model
Source: Front Aging Neurosci. 2018 Jun 26;10:187. doi: 10.3389/fnagi.2018.00187 (PMC6028736; doi:10.3389/fnagi.2018.00187)
Supplement: TABLE S4 — Taxonomic assignments of the 58 altered OTUs responding to oil treatments. [file Table_4.pdf]

**Supplementary Table S4.** Taxonomic assignments of 58 altered OTUs responding to oil treatment.

| OUT name | phylum | class | order | family | genus |
|---|---|---|---|---|---|
| OTU_06148 | *Bacteroidetes* | *Bacteroidia* | *Bacteroidales* | *Porphyromonadaceae* | *Barnesiella* |
| OTU_00124 | *Firmicutes* | *Clostridia* | *Clostridiales* | unclassified | unclassified |
| OTU_00022 | *Bacteroidetes* | *Bacteroidia* | *Bacteroidales* | *Porphyromonadaceae* | *Barnesiella* |
| OTU_00912 | *Bacteroidetes* | *Bacteroidia* | *Bacteroidales* | *Porphyromonadaceae* | *Barnesiella* |
| OTU_00215 | *Bacteroidetes* | *Bacteroidia* | *Bacteroidales* | *Porphyromonadaceae* | *Barnesiella* |
| OTU_00121 | *Firmicutes* | *Clostridia* | *Clostridiales* | *Lachnospiraceae* | *Clostridium XlVa* |
| OTU_00273 | *Firmicutes* | *Clostridia* | *Clostridiales* | *Ruminococcaceae* | *Cellulosibacter* |
| OTU_06159 | *Candidatus Saccharibacteria* | NA | NA | NA | *Saccharibacteria_genera_incertae_sedis* |
| OTU_00113 | *Firmicutes* | *Clostridia* | *Clostridiales* | *Lachnospiraceae* | unclassified |
| OTU_00889 | *Firmicutes* | *Clostridia* | *Clostridiales* | *unclassified* | unclassified |
| OTU_00245 | *Firmicutes* | *Clostridia* | *Clostridiales* | *Lachnospiraceae* | unclassified |
| OTU_00823 | *Firmicutes* | *Erysipelotrichia* | *Erysipelotrichales* | *Erysipelotrichaceae* | unclassified |
| OTU_00405 | *Firmicutes* | *Clostridia* | *Clostridiales* | unclassified | unclassified |
| OTU_00831 | *Firmicutes* | *Erysipelotrichia* | *Erysipelotrichales* | *Erysipelotrichaceae* | unclassified |
| OTU_00027 | *Proteobacteria* | *Gammaproteobacteria* | *Enterobacteriales* | *Enterobacteriaceae* | *Escherichia/Shigella* |

| | | | | | |
|---|---|---|---|---|---|
| OTU_00174 | *Firmicutes* | *Clostridia* | *Clostridiales* | *Lachnospiraceae* | *Ruminococcus2* |
| OTU_00411 | *Firmicutes* | *Clostridia* | *Clostridiales* | *Lachnospiraceae* | *Clostridium XlVa* |
| OTU_00098 | *Bacteroidetes* | *Bacteroidia* | *Bacteroidales* | *Rikenellaceae* | *Alistipes* |
| OTU_00294 | *Firmicutes* | *Clostridia* | *Clostridiales* | *Lachnospiraceae* | *Clostridium XlVa* |
| OTU_00328 | *Firmicutes* | *Clostridia* | *Clostridiales* | *Lachnospiraceae* | *Lachnospiracea_incertae_sedis* |
| OTU_00084 | *Bacteroidetes* | *Bacteroidia* | *Bacteroidales* | *Bacteroidaceae* | *Bacteroides* |
| OTU_00034 | *Actinobacteria* | *Actinobacteria* | *Bifidobacteriales* | *Bifidobacteriaceae* | *Bifidobacterium* |
| OTU_28470 | *Firmicutes* | *Bacilli* | *Lactobacillales* | *Lactobacillaceae* | *Lactobacillus* |
| OTU_00003 | *Firmicutes* | *Bacilli* | *Lactobacillales* | *Lactobacillaceae* | *Lactobacillus* |
| OTU_00006 | *Proteobacteria* | *Deltaproteobacteria* | *Desulfovibrionales* | *Desulfovibrionaceae* | *Bilophila* |
| OTU_00092 | *Bacteroidetes* | *Bacteroidia* | *Bacteroidales* | *Porphyromonadaceae* | *Odoribacter* |
| OTU_00103 | *Bacteroidetes* | *Bacteroidia* | *Bacteroidales* | *Porphyromonadaceae* | *Barnesiella* |
| OTU_00070 | *Bacteroidetes* | *Bacteroidia* | *Bacteroidales* | *Porphyromonadaceae* | *Barnesiella* |
| OTU_00180 | *Firmicutes* | *Bacilli* | *Lactobacillales* | *Lactobacillaceae* | *Lactobacillus* |
| OTU_00036 | *Firmicutes* | *Clostridia* | *Clostridiales* | *Lachnospiraceae* | *Clostridium XlVa* |
| OTU_00367 | *Firmicutes* | *Clostridia* | *Clostridiales* | *Lachnospiraceae* | *Acetitomaculum* |
| OTU_00126 | *Bacteroidetes* | *Bacteroidia* | *Bacteroidales* | *Porphyromonadaceae* | *Barnesiella* |

| | | | | | |
|---|---|---|---|---|---|
| OTU_00057 | *Firmicutes* | *Clostridia* | *Clostridiales* | *Ruminococcaceae* | *Ruminococcus* |
| OTU_00278 | *Firmicutes* | *Clostridia* | *Clostridiales* | *Lachnospiraceae* | *Clostridium XlVa* |
| OTU_00038 | *Firmicutes* | *Clostridia* | *Clostridiales* | *Lachnospiraceae* | *Lachnospiracea_incertae_sedis* |
| OTU_00037 | *Firmicutes* | *Clostridia* | *Clostridiales* | *Ruminococcaceae* | *Gemmiger* |
| OTU_01084 | *Bacteroidetes* | *Bacteroidia* | *Bacteroidales* | *Porphyromonadaceae* | *Coprobacter* |
| OTU_00048 | *Firmicutes* | *Clostridia* | *Clostridiales* | *Lachnospiraceae* | unclassified |
| OTU_00287 | *Bacteroidetes* | *Bacteroidia* | *Bacteroidales* | *Porphyromonadaceae* | *Barnesiella* |
| OTU_00349 | *Bacteroidetes* | *Bacteroidia* | *Bacteroidales* | *Rikenellaceae* | *Alistipes* |
| OTU_00477 | *Firmicutes* | *Clostridia* | *Clostridiales* | *Ruminococcaceae* | *Gemmiger* |
| OTU_06169 | *Firmicutes* | *Clostridia* | *Clostridiales* | *Lachnospiraceae* | *Clostridium XlVa* |
| OTU_01108 | *Planctomycetes* | *Planctomycetia* | *Planctomycetales* | *Planctomycetaceae* | *Aquisphaera* |
| OTU_00040 | *Firmicutes* | *Bacilli* | *Bacillales* | *Staphylococcaceae* | *Staphylococcus* |
| OTU_00526 | *Actinobacteria* | *Actinobacteria* | *Acidimicrobiales* | *Acidimicrobiaceae* | *Ilumatobacter* |
| OTU_00269 | *Firmicutes* | *Clostridia* | *Clostridiales* | *Ruminococcaceae* | unclassified |
| OTU_00407 | *Firmicutes* | *Clostridia* | *Clostridiales* | *Lachnospiraceae* | unclassified |
| OTU_02864 | *Bacteroidetes* | *Bacteroidia* | *Bacteroidales* | *Porphyromonadaceae* | *Tannerella* |
| OTU_06201 | *Bacteroidetes* | *Bacteroidia* | *Bacteroidales* | *Porphyromonadaceae* | *Barnesiella* |

| | | | | | |
|---|---|---|---|---|---|
| OTU_09375 | *Firmicutes* | *Clostridia* | *Clostridiales* | *Lachnospiraceae* | *Eisenbergiella* |
| OTU_00255 | *Firmicutes* | *Clostridia* | *Clostridiales* | *Lachnospiraceae* | unclassified |
| OTU_00390 | *Firmicutes* | *Erysipelotrichia* | *Erysipelotrichales* | *Erysipelotrichaceae* | unclassified |
| OTU_00347 | *Firmicutes* | *Clostridia* | *Clostridiales* | *Ruminococcaceae* | *Clostridium IV* |
| OTU_00063 | *Firmicutes* | *Bacilli* | *Bacillales* | *Staphylococcaceae* | *Jeotgalicoccus* |
| OTU_00074 | *Firmicutes* | *Erysipelotrichia* | *Erysipelotrichales* | *Erysipelotrichaceae* | *Allobaculum* |
| OTU_00323 | *Firmicutes* | *Clostridia* | *Clostridiales* | unclassified | unclassified |
| OTU_00031 | *Firmicutes* | *Bacilli* | *Bacillales* | *Staphylococcaceae* | *Staphylococcus* |
| OTU_00219 | *Actinobacteria* | *Actinobacteria* | *Actinomycetales* | *Corynebacteriaceae* | *Corynebacterium* |
