# Supplementary material for: Sex-Based Differences in Gut Microbiota Composition in Response to Tuna Oil and Algae Oil Supplementation in a D-galactose-Induced Aging Mouse Model
Source: Front Aging Neurosci. 2018 Jun 26;10:187. doi: 10.3389/fnagi.2018.00187 (PMC6028736; doi:10.3389/fnagi.2018.00187)
Supplement: TABLE S5 — Relative abundance of the 58 altered OTUs responding to tuna oil, algae oil and mixed treatment identified by RDA. [file Table_5.pdf]

Supplementary Table S5. Relative abundance of the 58 altered OTUs responding to tuna oil, algae oil and mixed treatment identified by RDA

OUT name	Control	D-gal	D-gal+D	TO600	AO600	TO200AO400
OTU_06148	1.250660071	0.002835753	0.003335557	0.003469572	0.000000000	0.005395489
OTU_00124	0.575303632	0.002835753	0.003335557	0.000000000	0.080620155	0.067443617
OTU_00022	1.73980712	0.011343013	0.000000000	0.638401221	0.613953488	1.362361066
OTU_00912	0.430782913	0.002835753	0.000000000	0.000000000	0.000000000	0.002697745
OTU_00215	0.642005503	0.005671506	0.890593729	0.000000000	0.000000000	0.000000000
OTU_00121	0.836552625	0.011343013	0.320213476	1.117202137	0.018604651	0.809323406
OTU_00273	0.842111114	0.017014519	0.016677785	0.256748317	0.15503876	0.070141362
OTU_06159	0.133403741	0.002835753	0.000000000	0.003469572	0.003100775	0.000000000
OTU_00113	0.653122481	0.014178766	0.143428953	0.010408716	0.024806202	0.064745872
OTU_00889	0.108390539	0.002835753	0.000000000	0.000000000	0.000000000	0.005395489
OTU_00245	0.528056474	0.014178766	0.026684456	0.031226147	0.052713178	0.008093234
OTU_00823	0.100052806	0.002835753	0.000000000	0.000000000	0.000000000	0.000000000
OTU_00405	0.097273561	0.002835753	0.000000000	0.000000000	0.012403101	0.005395489
OTU_00831	0.091715072	0.002835753	0.000000000	0.003469572	0.000000000	0.000000000
OTU_00027	0.311275395	3.567377495	0.747164777	0.274096177	8.173643411	0.992770044
OTU_00174	0.086156583	0.995349365	0.03335557	0.03816529	0.00620155	0.099816553
OTU_00411	0.002779245	0.034029038	0.003335557	0.003469572	0.000000000	0.005395489
OTU_00098	0.005558489	0.073729583	0.186791194	0.14919159	0.043410853	0.051257149
OTU_00294	0.002779245	0.036864791	0.000000000	0.013878287	0.000000000	0.002697745
OTU_00328	0.002779245	0.036864791	0.000000000	0.031226147	0.000000000	0.000000000
OTU_00084	0.077818849	1.239224138	0.020013342	0.33654847	0.021705426	1.278730981
OTU_00034	0.016675468	0.3573049	0.030020013	0.183887308	0.514728682	0.509873746
OTU_28470	0.002779245	0.068058076	0.003335557	0.000000000	0.000000000	0.002697745
OTU_00003	0.063922626	1.789360254	0.500333556	4.565956561	15.64031008	0.178051149
OTU_00006	0.005558489	0.246710526	0.000000000	0.294913608	0.049612403	0.696018129
OTU_00092	0.002779245	0.195666969	0.03335557	0.4683922	0.148837209	0.008093234

OTU_00103	0.005558489	0.422527223	0.170113409	0.388592048	0.291472868	1.184309917
OTU_00070	0.002779245	0.258053539	0.000000000	0.000000000	0.195348837	0.002697745
OTU_00180	0.152858453	0.045372051	0.000000000	0.010408716	0.136434109	0.312938384
OTU_00036	0.130624496	0.207009982	0.000000000	0.117965443	0.000000000	0.002697745
OTU_00367	0.152858453	0.000000000	0.000000000	0.006939144	0.000000000	0.000000000
OTU_00126	0.000000000	0.000000000	0.000000000	0.097148012	0.003100775	1.66990396
OTU_00057	0.058364137	0.00850726	0.070046698	0.388592048	0.096124031	0.105212043
OTU_00278	0.161196187	0.056715064	0.000000000	0.052043578	0.015503876	0.035070681
OTU_00038	0.033350935	0.045372051	0.000000000	0.000000000	0.102325581	0.024279702
OTU_00037	0.03613018	0.056715064	0.003335557	0.010408716	0.003100775	0.035070681
OTU_01084	0.030571691	0.351633394	0.026684456	0.024287003	0.220155039	0.110607532
OTU_00048	0.252911259	1.729809437	0.770513676	0.319200611	0.018604651	0.191539873
OTU_00287	0.013896223	0.000000000	0.010006671	0.000000000	0.000000000	0.000000000
OTU_00349	0.011116978	0.042536298	0.003335557	0.079800153	0.046511628	0.032372936
OTU_00477	0.002779245	0.017014519	0.000000000	0.000000000	0.003100775	0.000000000
OTU_06169	0.000000000	0.000000000	0.000000000	0.003469572	0.000000000	0.013488723
OTU_01108	0.013896223	0.000000000	0.010006671	0.010408716	0.018604651	0.002697745
OTU_00040	0.080598093	0.087908348	0.040026684	0.013878287	0.046511628	0.032372936
OTU_00526	0.052805647	0.014178766	0.026684456	0.031226147	0.024806202	0.018884213
OTU_00269	0.069481115	0.014178766	0.003335557	0.027756575	0.024806202	0.000000000
OTU_00407	0.005558489	0.113430127	0.003335557	0.003469572	0.003100775	0.002697745
OTU_02864	0.000000000	0.002835753	0.000000000	0.104087156	0.052713178	0.000000000
OTU_06201	0.000000000	0.000000000	0.000000000	0.000000000	0.000000000	0.008093234
OTU_09375	0.000000000	0.000000000	0.000000000	0.000000000	0.000000000	0.000000000
OTU_00255	0.005558489	0.005671506	0.060040027	0.041634862	0.000000000	0.037768426
OTU_00390	0.002779245	0.002835753	0.053368913	0.000000000	0.003100775	0.005395489
OTU_00347	0.041688669	0.002835753	0.000000000	0.013878287	0.000000000	0.048559404
OTU_00063	0.041688669	0.025521779	0.023348899	0.052043578	0.024806202	0.024279702
OTU_00074	0.130624496	0.283575318	0.003335557	0.079800153	0.133333333	1.065609151

OTU_00323	0.169533921	0.000000000	0.006671114	0.364305045	0.018604651	0.013488723
OTU_00031	0.219560324	0.204174229	0.110073382	0.315731039	0.502325581	0.091723319
OTU_00219	0.047247158	0.019850272	0.026684456	0.180417736	0.024806202	0.043163915
